# Health of the Empire

**Published:** 1924-07

**Authors:** 


### THE HEALTH OF THE EMPIRE.

Knowledge, clearness of mind, the broad vision, strength of will and sympathy of heart have been in the past, and they will be in the future, the inspiration of all high human endeavour. Writing thus a few years ago, Sir George Newman avowed his belief that the science and art of preventive medicine need the same inspiration. Though we cannot all be public health pundits, our interest can be aroused in, and knowledge thereby gained of, what has been accomplished in the past, so that, slowly it may be and unconsciously, we may help to form that public opinion which is essential to true progress. Happily, there is a never-failing supply of men with clearness of mind and wide vision to point the way. In this country we have trodden the path of sanitary progress begun in the Victorian period, and have made much headway. We have almost taken to heart the gospel of soap and water. We have more recently seen tremendous advances in bacteriology; the man with the microscope has wrested valuable secrets from nature. We have especially learned valuable lessons in the Great War. The public generally need more and more enlightenment in the form of a history of what has been accomplished, and in records simply told in a manner not dulled by statistics or scientific detail, of the work of those who carry on the fight against disease.

In the present year of grace we are not allowed to forget, if we would, that we are heirs to a vast and mighty Empire. There comes into our hands opportunely a book which we review elsewhere, which very worthily deals with the subject of public health as a vital factor in the welfare of that Empire. We are reminded that our colonies are struggling in different parts of the world with problems that have been our own in greater or less degree, and benefiting by our experience; at the same time they are tackling questions peculiarly their own from which we in turn shall benefit. We are told in particular that there is no hard and fast line between home hygiene and hospital hygiene. The advance of each is dependent on research and education. In regard to research, there is some approach to equality of opportunity in the tropics and at home. When we turn to education and realise what an uphill task in this country it has been and still is to awaken the public conscience and form an enlightened public opinion, what are we to think of the difficulties of those who work among native populations? Who is to preach the value of the open window at night to a West Indian negro who lives in terror of "duppies," or to try and get the Hindu to grasp the significance of the germ theory of communicable disease while he worships strange deities from whom he believes sickness and death emanate?

In the process of our own education at home such a book as this on the health problems of the Empire should play an invaluable part; for the schoolroom where is being moulded public opinion of to-morrow it has a wealth of lessons. If we will only grasp the facts we shall, without doubt, strengthen or force, as the case may require, the hands of our administrators, and they will go forward where the pioneer and the worker in the field of research are always leading, so that in every corner of our Empire the headway which is being made against dirt and disease may be a source of wonder and inspiration to the rest of the world. And let us remember that fine words butter no parsnips, and see to it that when we are asked to pay the cost of preventive measures we do so cheerfully in the consciousness that sound health is a safe investment.

